# Breast ultrasound in high-risk women

**DOI:** 10.18632/oncotarget.26713

**Published:** 2019-02-22

**Authors:** Laura Cortesi, Elena Barbieri, Angela Toss

**Affiliations:** Department of Oncology and Haematology, Genetic Oncology Unit, University Hospital of Modena, Modena, MO, Italy

**Keywords:** breast ultrasound, high risk

Ultrasound (US) has traditionally been used as a mammography (MMG) support in diagnostic tool for detection of invasive breast cancer (BC), particularly in young women and with dense breast where the US sensitivity is very high (95.7%) [1-2]. An advantage of US, is that it does not use ionizing radiation so can be reserved for BRCA mutation carriers [3]. The American College of Radiology and the Society of Breast Imaging recommend adding US approach to MMG only in a very subgroup of people with a strong hereditary risk (>20% lifetime risk) and/or with contraindications to MRI [4]. However, the benefit in women with a familial risk has not been established yet.

In our previous work, four different risk categories were defined according with Modena criteria and Gail model calculation: BRCA carriers, high risk (HR) group with a life-time risk cut-off of 30-50%, intermediate risk (IR) group (life-time cut-off ranging between 18 and 29%) and slightly increased risk (SIR) group (less than 18%) [5]. Since we showed that the standardized incidence ratios were significantly higher in all but the SIR group, in our recent publication we evaluated the contribution of US screening in detecting BC among only BRCA carriers (136 women), HR (1749 women) and IR groups (428 women), both in association with MMG and with semi-annual clinical breast examination, finding that combined sensitivity for MMG plus US was 100% in HR and 80.4% for IR women (p < 0.01). In BRCA mutated patients, breast magnetic resonance imaging (MRI) alone provided 94% sensitivity, reaching 100% in patients older than 50 years by adding annual MMG and biannual US.

At the US diagnosis, we found mostly ductal infiltrating carcinoma compared with in situ carcinomas (DCIS) (82.7% vs. 17.3%), but the DCIS rate was significantly increased in the IR group. This finding justifies the evidence that patients at intermediate risk develop less aggressive tumors than patients at high risk or BRCA carriers. The US diagnosed tumor size was less than 2 cm in 86% of cases, demonstrating that US is able to detect BC at early stage in the most of cases. Once again, cancers diagnosed by US in BRCA carriers were mostly triple negative BC, with high grade. Of interest, the US sensitivity was a little bit decreased after 50 years (31.5% in patients aged ≤ 50 years vs. 27.7 % in patients older than 50 years), when breast density become more adipose [6]. Also Brem et al. used three-dimensional US in a generalizable cohort of women with dense breasts, showing that it improves the detection rate of mammographic screening, but also increases the number of false-positive results [7].

In our experience the real US benefit was represented by the six-monthly examination reaching a 29.4% of sensitivity, without significant differences amongst the risk groups and age range. The US sensitivity in BRCA carriers was 22.7%, but in that population breast MRI alone represented the most sensitive technique (94%). For patients belonging to the HR, our schedule and timing based on six-monthly US starting at 30 years, biennial mammogram from 30 to 36 years, annual from 36 years on, should be referred as screening protocol, since provide a total sensitivity of 100%. On the other hand, in the IR group, particularly for women aged more than 50 years, the US sensitivity was very low (18.2%).

We think that in women at high risk without BRCA mutations is justified to enhance the screening by US, since it is a safe technique at low costs. This message is very important for women who are the most considerable part of the population at risk and for which standard protocol screening do not provide sufficient sensitivity in BC diagnosis. For women at IR, annual mammogram without ultrasound has probably sufficient sensitivity. Women with BRCA mutation have an advantage in BC diagnosis by MRI even if a mortality reduction have not been yet demonstrated.

We suggest that breast US can be a useful addition to mammogram for BC screening, in certain cases. A correct identification of different BC risk levels is needed to provide several screening protocols, in which US could be added without significant additional costs and reducing the incidence of interval breast cancer with early diagnosis.

Results from the ongoing HIBCRIT3 study comparing breast annual MRI plus US after 6 months vs. annual MRI plus MMG and US after 6 months in BRCA1/2 carriers, are awaited to provide further evidence, in order to improve the screening of women with mutations in hereditary breast cancer genes.
